# Prognostic and biologic significance of high LCK expression in ovarian cancer

**DOI:** 10.1186/2051-1426-3-S2-P93

**Published:** 2015-11-04

**Authors:** Amir Jazaeri, Cherie Paquette, Sarah M Kelting, Mark H Stoler, Qian Zhang, Melinda Yates, Caitlin Creasy, Chantale Bernatchez, Patrick Hwu

**Affiliations:** 1 UT MD Anderson Cancer Center, Houston, TX, USA; 2 Women and Infants Hospital/The Warren Alpert School of Medicine of Brown University, Providence, RI, USA; 3 University of Virginia, Charlottesville, VA, USA

## Introduction

Epithelial ovarian cancer is the most lethal and second most common gynecologic cancer in the United States. Previous studies have demonstrated the favorable prognostic value of tumor infiltrating lymphocytes in this disease. The objective of this study was to evaluate the prognostic value and the biological correlates for lymphocyte specific kinase (LCK) expression in high grade serous ovarian cancer.

## Methods

LCK mRNA and protein (included in the reverse Phase protein array (RPPA) data) expression was evaluated using the ovarian cancer TCGA dataset. Gene expression was compared between the LCK high and normal tumors was compared. LCK protein expression was also evaluated using IHC in an independent set of high grade serous ovarian cancers, borderline tumors, benign cystadenomas, and normal fallopian tubes (tissue of origin of serous neoplasms).

## Results

Using a SD of 1.86 above the mean as a cutoff, LCK mRNA high samples were associated with both an improved progression free and overall survival (p

## Conclusions

LCK serves as a biomarker of prognostic and biological importance in ovarian cancer. Ongoing investigation is aimed at better understanding the immunological correlates of high LCK expression in ovarian cancer.

